# Supplementary material for: Applying machine learning and geolocation techniques to social media data (Twitter) to develop a resource for urban planning
Source: PLoS One. 2021 Feb 3;16(2):e0244317. doi: 10.1371/journal.pone.0244317 (PMC7857609; doi:10.1371/journal.pone.0244317)
Supplement: S1 File — (PDF) [file pone.0244317.s001.pdf]

# Applying Machine Learning and Geolocation Techniques to Social Media Data (Twitter) to Develop a Resource for Urban Planning

## Supplementary Information

The findings, interpretations, and conclusions expressed in this paper are entirely those of the authors. They do not necessarily represent the views of the World Bank and its affiliated organizations, or those of the Executive Directors of the World Bank or the governments they represent.

In recent years, social media, and especially Twitter, has emerged as a source of real-time information. Therefore, it is natural that in a context where there is a dearth of official data on a topic such as road safety, we turn to crowdsourcing through Twitter to produce more comprehensive information on road traffic crashes. As people move around cities that have been plagued by congestion, they have started to rely on social media and citizen reporting to help them avoid major traffic jams and decrease their commutes. Given the relationship between RTCs and congestion, platforms that crowdsource and broadcast traffic updates have the additional benefit of often reporting on RTCs. This makes it possible to use crowdsourced data to identify when and where crashes are occurring, which can be used to supplement and improve on existing official statistics.

While only around 1% of tweets contain geo-metadata, a growing literature has developed geoparses—or algorithms that extract location names from text. Tweets present a unique challenge to geoparses. State-of-the art geoparses, such as OpenCascais and Stanford Named Entity Recognition, rely on grammar rules to identify location mentions; however, tweets often do not follow grammatical capitalization rules and use clipped, ungrammatical sentences [1, 2]. New algorithms have been developed to geoparse tweets. This includes developing an algorithm that accounts for tweets that contain place references that are abbreviated, misspelled or highly localized [2]. Others develop gazetteers (location dictionaries) from sources such as Open Street Map and Geonames and search for names within the gazetteer in tweets, employing different approaches to account for misspellings or tweets using shortened names than are in gazetteers [3, 4, 5]. [5] provides a review of existing approaches.

Here we provide more information on the specific data that we used, how they were processed and the algorithms that were developed. These processes can then be implemented in different contexts where crowdsourced data on RTCs are available.

## Twitter Data

We generate crash data from social media. The main source of crowdsourced data comes from Ma3Route, a mobile/web/SMS platform that crowdsources transport data and provides users with information on traffic, matatu (informal bus) directions, driving reports and crashes for Kenya. As of early 2019, Ma3Route had 1.1 million followers on Twitter and around 400,000 subscribed users on their app. When users post a traffic report on the app, Ma3Route displays the report on their

app and posts the report to Twitter. We scraped all tweets posted by Ma3Route from May 2012, when the Twitter feed was started, onward. Figure S1 shows the number of tweets across time.[1] The full dataset of tweets that we use consists of 874,588 tweets scraped between May 2012 and July 2020. See Table S1 for examples of tweets.

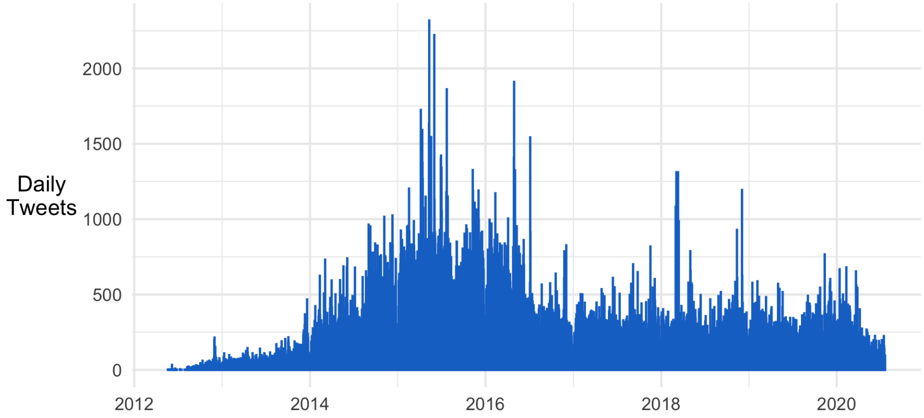


Figure S1: Ma3Route Tweet Trends

Table S1: Example tweets from Ma3Route

| | |
|---|---|
| 1 | accident on waiyaki way just before deloitte |
| 2 | accident just before roysambu footbridge on thika road inboud tailback almost at githurai |
| 3 | accident at junction of ole dume and arwing kodhek |
| 4 | accident at tajmall towards the roundabout heavy traffic from doni |
| 5 | there is an accident at the pangani underpass heading to either muranga road or forest road involving two personal cars and a matatu mini bus this is causing a bit of snurl up cc |
| 6 | jogoo road traffic small accident just before donholm |
| 7 | a heavy truck has rolled at karai naivasha loaded with what seems to be bags of maize such trucks are supposed to use mai mahiu route how did it end up there |
| 8 | prepare for snurl up jogoo road just a minor incident apo hamza |
| 9 | bad accident involving 6 matatus and a lorry on thika road near till station |
| 10 | an accident has occurred kenyatta road involving a lorry that has overturned and several vehicles |

User mentions have been removed.

[1] Ma3Route was most popular in 2015, receiving 700-1,000 traffic reports a day, but has since declined in popularity, receiving an average of around 300 traffic reports daily.

We explored additional Twitter handles that focus on traffic and road safety in Kenya. These include twitter handles such as RoadAlertsKE, KenyanTraffic and ThikaTowntoday. The majority of tweets from these other handles are already tweeted out by Ma3Route; therefore, including these additional handles does not produce many new tweets to incorporate into the dataset. An additional source of data is including tweets that mention Ma3Route but are not necessarily posted by Ma3Route. While these tweets are not included in the current analysis, they can be easily incorporated to expand the data set that is used to generate additional crash reports. We have already done this for the data set of crashes that we are producing for the Government of Kenya.

### Building a Truth Data set of Tweets

We build a truth data set of Ma3Route tweets where tweets are labeled as to whether they refer to a specific traffic crash and, if they do, are geocoded. We code all potentially crash related tweets from July 2017 to July 2018. We define a tweet as potentially crash-related if one of the following words appeared in the tweet:

*accident, accidents, ajali, axident, collision, crash, crashes, crashs, crush, crushed, damage, disaster, emergency, fatal, fatality, fender bender, fender-bender, hazard, hit, hit-and-run, incident, incidents, injuries, injury, magari zmegongana, mishap, overturn, overturned, ovrturn, ovrturned, pileup, rammed, read end, rear ended, roll, rolled, smash, smashed, wreck, wreckage, zilicrash, zime-crash*

To account for misspellings of select words, we also include tweets if they contained a word that had a Levenshtein distance of two or less to "accident" or "incident" or a Levenshtein distance of one to "crash" or "crashed".

Six coders were trained to process the 9,480 tweets defined as potentially crash related. Coders were instructed to label a tweet as reporting a crash if the tweet referred to one or more specific crashes; general comments about crashes were labeled as not reporting a crash. If the coder labeled the tweet as reporting a crash, they were instructed to geocode the location of the crash based on the tweet text if they were able. Coders were instructed to record the street names and landmarks used to geocode the crash. In addition, they provided the approximate coordinates of the crashes.

Each tweet was labeled and geocoded by two coders; differences were resolved by one of the authors. (We consider geocodes different if they were more than 100 m apart.)

Of the 9,480 tweets, 6,602 (69%) reported a crash and of these, 4,192 (63%) identified an approximate location of the crash.

## Augmenting a Gazetteer

The primary goal of the algorithm to augment the gazetteer is to generate alternate names of landmarks that users may use instead of the original name in the gazetteer. Alternate names are generated in three steps: (1) splitting landmark names at certain punctuation (e.g., slashes), (2) create n-grams and skip-grams of landmarks and (3) in select cases, removing the landmark type from the end of the name (e.g., removing 'restaurant' from 'McDonald's restaurant.') The algorithm also removes landmark names that are common words that may often be used in a context to not refer to a landmark. In addition, the algorithm removes landmarks that do not refer to a specific location, such as roads.

| **Algorithm** | Augment gazetteer |
|---|---|
| **Input** | Landmark gazetteer, where for each landmark entry includes: (1) name, (2) types and (3) coordinates |
| **Output** | Augmented landmark gazetteer |

**A. Split landmarks at select punctuation**

1. If a landmark has a slash, open parentheses, dash or comma, add landmarks to the gazetteer that separate at the character.

---

**B. Clean landmark names**

1. Everything lowercase, only keep alphanumeric characters (eg, remove punctuation)

---

**C. Remove certain landmarks**

1. Remove landmarks that are just one character in length
2. Remove landmarks that have certain types (eg, where the type indicates that the landmark actually represents a large area). We remove landmarks with the type: route, road, political, locality or neighborhood except if the landmark also contains "flyover" or "roundabout" in the name[1]

---

**D. Create N-grams and skip-grams[2]**

1. Generate 2-3 N-grams and add to gazetteer
2. Generate 2-3 skip-grams, skip 1-4, restrict so that the first and last word match and add to gazetteer[3]

---

**E. Create parallel landmarks**

1. If a word begins/ends with a certain word/phrase, remove the word or phrase
   (a) If it begins with a stopword or preposition, create parallel landmark with word removed
   (b) If ends with: bar, shops, restaurant, hotel, stage, bus stop or bus station, create parallel landmark with word removed
2. If word contains certain word/phrase, swap with another
   (a) (stage, bus stop, bus station) – make interchangeable. So if someone says "X stage", create "X bus stop" and "X bus station"
3. Different spellings of words
   (a) British/English spellings (Eg,: center vs centre, theater vs theatre)

[1] We treat flyovers and roundabouts as landmarks, even though they are roads, as they represent a unique location

[2] Other geoparses such as LNEx only add the n-grams and skip-grams if the name does not already exists in the gazetteer. Our algorithm differs, and we add all n-grams and skip-grams. However, in the algorithm to locate events, we preference locations where the landmark name associated with the location was not a derived n/skip-gram, but still consider the n-gram/skip-gram version as the non-derived landmark location may be removed from consideration if it is not near a mentioned road.

[3] For example, from the original landmark 'Prestige Plaza Shopping Mall', this generates 'Prestige Mall', 'Prestige Plaza Mall', and 'Prestige Shopping Mall'

   (b) Common shorter/longer/different ways (train vs railway, rail vs railway)

4. Add types
   (a) If landmark ends with: stage, bus stop or bus station, add "stage" as type (we preference certain types, hence we do this).
5. Remove parallel landmarks if only 1-2 characters long, and add rest to gazetteer

---

**F. Remove landmarks**

1. If it has a stop word and is 2 or less words, remove
2. If landmark contains/begins with/ends with:
   (a) If landmark contains: road or rd, remove
   (b) If landmark begins with a stop word or preposition, remove
   (c) If landmark ends with road word (street, st, avenue, ave), remove
3. Remove common English words
   (a) Remove one word landmarks that are also English words (spelled correctly according to an English spell checker)[4] but are not nouns[5] or categorized as a bus/ transit station.[6]

---

[4]We use Hunspell, a commonly used spellchecker

[5]We use spaCy, an open source natural language processing library, to determine the part of speech of each landmark

[6]We keep bus/transit stations as users often reference matatu stages when describing crash locations

## Tweet Classification - Identifying relevant crowdsourced reports

We first developed an algorithm to identify whether a tweet is crash related or not, using the truth data set to train the algorithm. We extract features from tweets by extracting n-grams from tweets. We employ a grid search, tuning the models by testing all combinations of multiple parameters. The three main parameters we test are: (1) extracting 1-grams, 1-2 grams or 1-3 grams, (2) not removing any features or removing features that occur in less than/more than 0.01%/99.9%, 1%/99% or 5%/95% of tweets, (3) defining features as the number of occurrences of the n-gram in the tweet or using the Term Frequency - Inverse Density Frequency (TF-IDF) of the n-gram[2] For the Support Vector Machine, we also vary the regularization parameter–which controls how the algorithm weighs misclassification versus simplicity–using 0.5, 1, 2, 10, 100 and 1000.

Table S2: Example Tweet and Augmented Tweet

| |
|---|
| accident past garden city near thika rd and kamiti rd junction |
| accident past #landmark-name# near #road-name# and #road-name# junction |

An additional parameter we test is using the original tweet text and, following [6], replacing landmark names and road networks with generalized names (just indicating the presence of a landmark or road). Generalizing landmark and road names helps to reduce the dimensionality of the feature space. Table S2 demonstrates how a particular tweet is transformed into one with general landmark and road names. Table S3 shows examples of the features extracted in regular and augmented tweets where landmarks and roads have been replaced. This augmentation assumes that the occurrence of a road or landmark name contributes equally to the probability of a crash-related tweet.

[2]TF-IDF reflects how important a word or n-gram is to a tweet within the full set of tweets; for example, words such as 'a' or 'the' that appear frequently will be given less weight. It is calculated as

$$log(\frac{N\ Tweets}{N\ Tweets\ with\ N-gram}) \times \frac{N\ times\ n-gram\ appears\ in\ atweet}{N\ n-grams\ in\ a\ tweet}$$

Table S3: Features of Tweets

| N-gram | Using Original Tweet | Using Augmented Tweet |
|---|---|---|
| accident | 1 | 1 |
| past | 1 | 1 |
| garden | 1 | 0 |
| city | 1 | 0 |
| near | 1 | 1 |
| thika | 1 | 0 |
| rd | 2 | 0 |
| and | 1 | 1 |
| kamiti | 1 | 0 |
| junction | 1 | 1 |
| accident past | 1 | 1 |
| past garden | 1 | 0 |
| garden city | 1 | 0 |
| city near | 1 | 0 |
| near thika | 1 | 1 |
| thika rd | 1 | 0 |
| rd and | 1 | 0 |
| and kamiti | 1 | 0 |
| kimiti rd | 1 | 0 |
| rd junction | 1 | 0 |
| #landmark-name# | 0 | 1 |
| #road-name# | 0 | 2 |
| past #landmark-name# | 0 | 1 |
| of #road-name# | 0 | 1 |
| and #road-name# | 0 | 1 |

Features defined using the number of occurrences of n-gram in the tweet.

We test two methods for determining whether a tweet reports a crash: Naive Bayes and support vector machines. Both techniques are commonly used in text classification for their ability to handle high dimensionality, e.g. when the number of features is greater than the number of observations [7, 8]. The Naive Bayes model is estimated as:

$$\hat{y} =_y P(y) \prod_{i=1}^{n} P(x_i|y) \tag{1}$$

where $y$ is whether the tweet is classified as crash related or not and $x_i$ are all the n-grams that occur in a tweet.

The linear SVM solves the minimization problem:

$$min\ C \sum_{i}^{N} (1 - y_i f(x_i))^2 + ||w||^2 \tag{2}$$

where C is a regularization parameter and $||w||^2$ is a penalty function. Here, $y$ equals 1 when the tweet references a crash and -1 when it does not. We use a squared hinge loss function (L2).

We implement k-fold cross-validation on 4 folds, training the model on 75% of the truth data and testing on 25% of the data within each fold. Table S4 shows results for select parameters. While the Naive Bayes algorithm performs slightly better based on precision, the SVM has higher recall and generally performs better for 2 and 3 n-grams. Overall, the F1 statistic, which provides a balance between the precision and recall, is best for SVM at 0.95 using 2 and 3-grams. Given that the overarching goal is to produce a data set of geolocated crashes based on the tweets, better recall is more important than higher precision. The reason for this is that even if a larger set of tweets is misclassified as crash related, it is more likely that these general tweets will not be geolocated at the second stage since they are not discussing a particular crash with a given location. We therefore want to capture as many of the tweets that are reporting crashes as possible at this stage, even if it means capturing slightly more tweets that are not reporting a crash. The SVM algorithm also has a very high accuracy of 0.93.

Table S4: Tweet Classification Results

| Precision | Recall | F1 | Accuracy | N-Grams |
|---|---|---|---|---|
| Naive Bayes | | | | |
| 0.938 | 0.947 | 0.942 | 0.919 | 1 |
| 0.945 | 0.949 | 0.947 | 0.926 | 2 |
| 0.945 | 0.949 | 0.947 | 0.926 | 3 |
| SVM | | | | |
| 0.935 | 0.963 | 0.948 | 0.927 | 1 |
| 0.94 | 0.966 | 0.953 | 0.934 | 2 |
| 0.939 | 0.967 | 0.953 | 0.934 | 3 |

The table shows best results for both SVM and Naive Bayes. For these results, both models use the original tweet and no features are removed. The Naive Bayes models do not use TF-IDF, while the SVM models do.

## Preparation for Geolocation

Prior to being able to use the geolocation algorithm, two additional pieces need to be prepared. One relates to identifying types of landmarks that are more common to be mentioned as the location of a crash in a tweet. In the situation where there might be multiple landmarks with the same name, the more likely landmark for a crash is the one that should be chosen for the location. The second relates to identifying the correct location when multiple locations are mentioned in the tweet. We can use the typical grammatical structure of a tweet to identify prepositions that are used prior to the correct location of a crash compared to ones that are more likely to be used with locations that are not close to the crash. Ranking prepositions based on these probabilities makes it possible to choose the correct location from the possible locations mentioned.

### Determining Landmark Types More Commonly Used as the Crash Location

When a landmark name is mapped to multiple locations, the algorithm preferences certain landmark types. To determine which landmarks to preference, we examine which landmark types are more commonly associated with the correct location. We consider cases where (1) one landmark is used to identify the crash location and (2) the landmark name is mapped to locations both near and far from the crash location. We compute the proportion of times a type is near and far from a crash location and divide the proportion near over far to understand the likelihood that choosing the type is near the crash location.

Figure S2 shows results. Among tweets considered, a landmark location that is a bus stop is near the correct location 17% of the time and is far from the correct location less than 1% of the time, leading to a bus stop being close to the correct location 22 times more frequently than far from the correct location.

In the algorithm, we use the top 6 landmark types (all being 2.5 or more times likely to be near the correct location) to preference landmarks: bus stop, parking, mall, cafe, transit station and bus station.

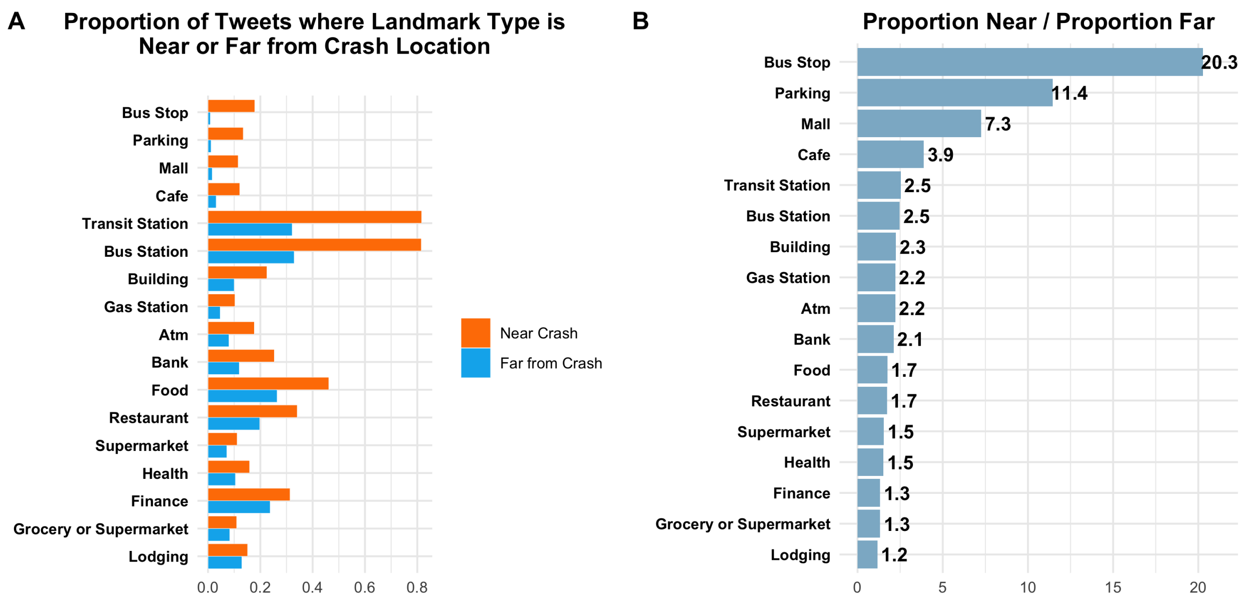


Figure S2: Landmark types typically near or far from the crash location when a landmark name is mapped to multiple locations

## Determining Preposition Phrase Tiers

The truth dataset indicates the landmark used to geocode the crash. We examine the phrases that precede the landmark. Figure S3 shows the top phrases. The phrase "at" precedes the correct landmark in 42% of tweets and in roughly half these cases "accident at" precedes the landmark.

We examine the phrases that precede the landmark to guide decision making when more than one landmark is mentioned. For this, we take all phrases that precede the correct landmark at least 20 times. We then identify cases where two of these phrases appear in a tweet and one of the phrases precedes the correct landmark; we then calculate the proportion of times each phrase precedes the correct landmark when the other phrase is also in the tweet. Figure S4 shows results. While 'at' is the most common word that precedes a landmark, other phrases that precede landmarks are more predictive of the correct landmark. For example, when both 'at' and 'near' appear in the tweet (and one of them precedes the correct landmark), the landmark is preceded by 'at' only 6% of the time. We use information from these phrase-pairings to divide phrases into "tiers"; if two landmarks are found in a tweet, the landmark is used where the phrase that precedes it is from a lower tier. We develop 6 tiers:

1. **Tier 1:** Across phrase-pairs, these phrases precede the correct landmark more than the other phrase in all cases. (for example, when ‘just after‘ and phrases such as ‘at’, ‘on’, or ‘in’ are also in the tweet, ‘just after‘ precedes the correct landmark more often than all other phrases).
2. **Tier 2:** These phrases precede the correct landmark more than the other phrase in over 90% of cases (but less than 100%).
3. **Tier 3:** Across phrase-pairs where one of the phrases is “at”, these phrases precede the correct landmark more times than “at.”
4. **Tier 4:** The phrase “at”
5. **Tier 5:** Remaining phrases where, across phrase-pairs, the phrase precedes the correct landmark more often than over half of the other phrases.
6. **Tier 6:** Remaining phrases where, across phrase-pairs, the phrase precedes the correct landmark more than at least one other phrase.

We modify this list to account for different spellings of certain phrases (e.g., adding “btw” with “between”) and whenever a phrase has “accident [word]”, we generalize so this becomes “[crash word] [word]”, where crash word includes any word such as accident, crash, hit, wreck, etc. Using this, we use the following phrase tiers:

1. **Tier 1:** [crash word] after, [crash word] near, [crash word] outside, [crash word] past, around, hapo, just after, just before, just past, near, next to, ”opposite”, outside, past, you approach, apa, apo, hapa, right after, right before, right past, just before you reach
2. **Tier 2:** [crash word] at, before
3. **Tier 3:** after
4. **Tier 4:** at, happened at, at the, pale
5. **Tier 5:** between, from, btw, btwn
6. **Tier 6:** along, approach, in, on, opp, to, towards, toward

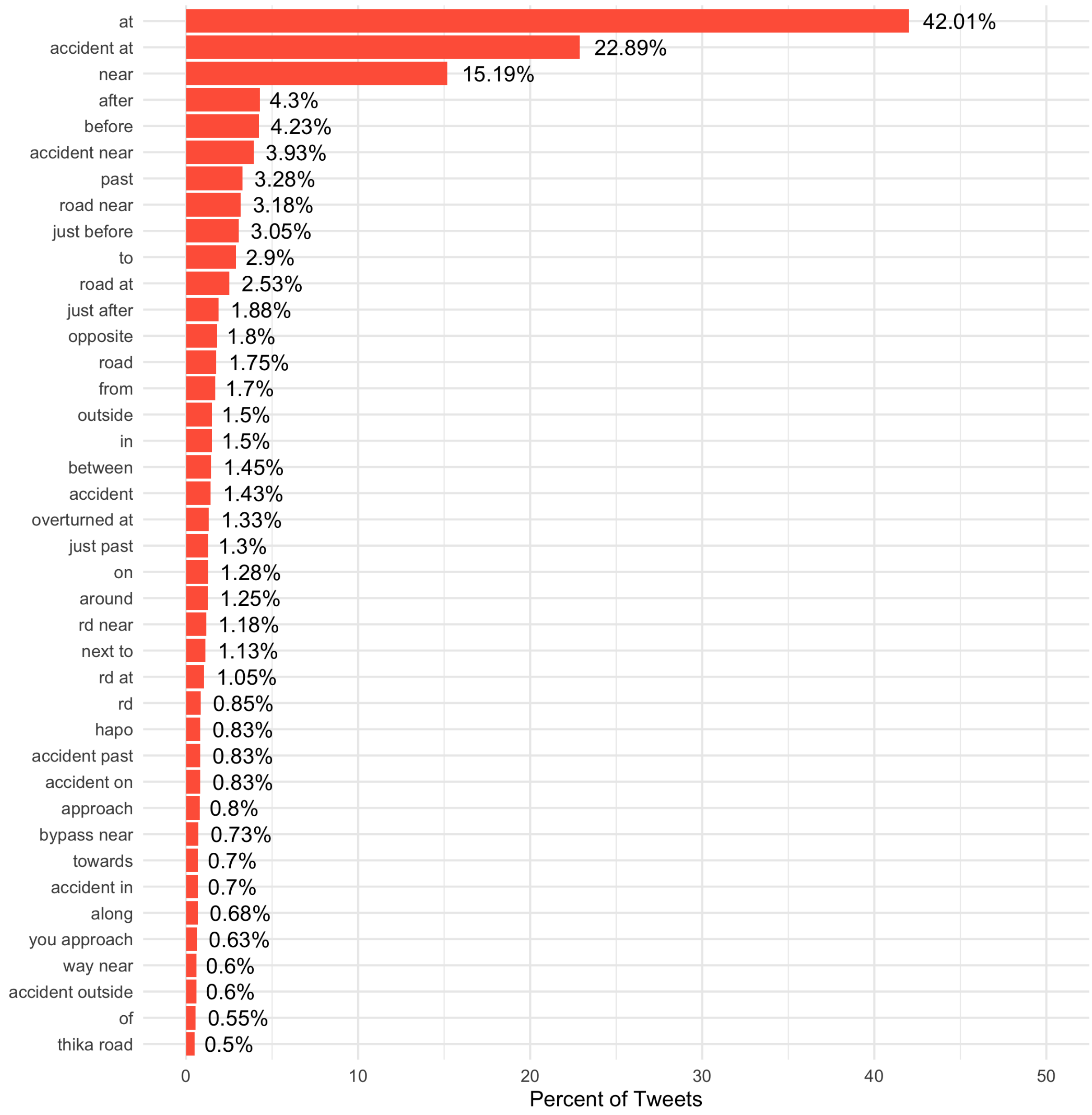


Figure S3: Top words that precede the landmark that correctly identifies the crash location.

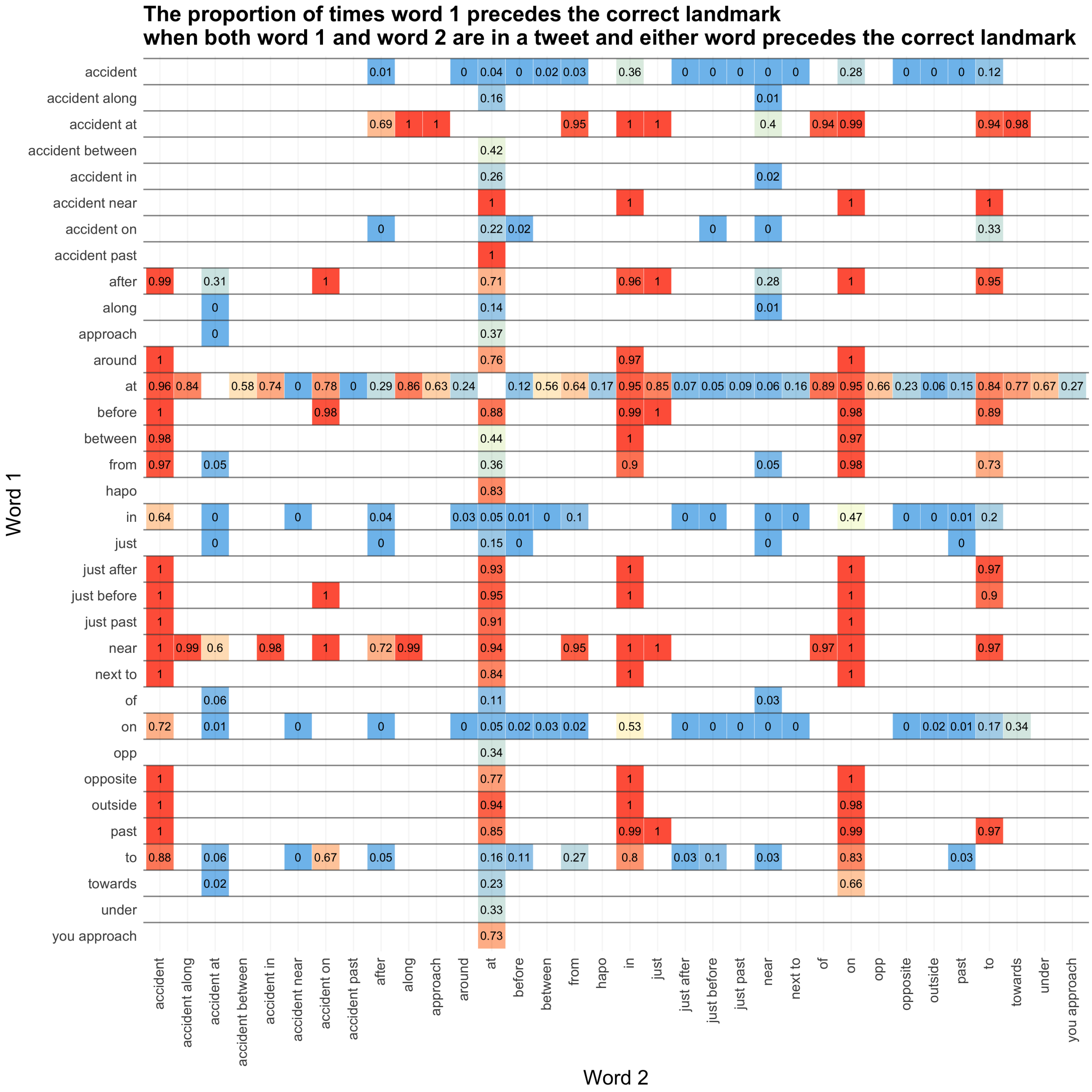


Figure S4: Likelihood of different words preceding the correct landmark

## Locating Crash Events

As demonstrated in Table S1 in the example tweets from @Ma3Route, the geoparser has to handle different tweets in different ways. For example, tweet 1 is simple, including the name of one road and one landmark. Tweet 3 is short and clear as well; however, it identifies the crash location by a junction instead of a landmark. Tweet 8 uses the Swahili word ”apo”, which is commonly in front of a landmark word. Accident 2 includes the location of the crash and the location where traffic starts. This section outlines in detail the different components of the geolocation algorithm, which are meant to handle these different situations.

The algorithm to locate an event location from text starts by cleaning the text and extracting location names of landmarks, roads and areas (e.g., neighborhoods) from the text. Next, the algorithm restricts location names and their locations to consider; for example, if two landmark names are found, and one is contained within the other, we only keep the longer one; in addition, where possible, we restrict locations to those near mentioned roads. The algorithm then chooses the location names that reference the event location, prioritizing location names primarily by the words that precede them (e.g., “just after [location]” is used over “toward [location]”). If the chosen location is not near a mentioned road, we search for landmarks that have a similar name but are near a mentioned road. Next, we snap the location to the road network. Finally, the algorithm implements select checks to determine whether no location should be outputted; for example, if a road is mentioned but the chosen location is not within 500 m from any mentioned road, the algorithm does not output a location. The algorithm is described in detail below.

| **Algorithm** Locate crash/event locations | |
|---|---|
| **Input** | Text |
| | Landmark gazetteer |
| | Roads |
| | Areas (e.g., neighborhoods) |
| | List of event words (e.g., crash, accident, wreck, etc) |
| | Prepositions, grouped by tier |
| | Types, grouped by tier |
| **Output** | Coordinates of event |

**A. Clean Tweets**

1. Replace @ with "at" only when it is not proceeded by via or when it is not the last word in a tweet.[7]
2. Remove select stopwords[8]
3. Mask common phrases that contain a location but refer to something else, such as "[city] bus"[9]
4. Removing hyperlinks and only keeping alphanumeric characters (e.g., removing punctuation).

---

**B. Extract Locations**

1. Extract exact matches of landmarks, roads and areas
2. Extract fuzzy matches of landmarks, roads and areas
   (a) Break tweets into 1-3 grams
   (b) For each n-gram, check levenstein distance to gazetteer entries. If word/phase is $0-4$ characters, ignore; if 5-10, allow levenstein distance of 1; if above 10, allow levenstein distance of 2
3. Extract landmarks after prepositions. For each preposition in the tweet:[10]
   (a) Take the word after the preposition and extract all landmarks that start with that word

[7] We found that @[word] often referred to a twitter handle when proceeded by via or when it was the last word in a tweet; otherwise, users were more likely to use "@" as a shorthand for "at." Distinguishing these cases is important as we rely on preposition to prioritize landmark references.

[8] We only remove "a" and "the"; other stopwords may be part of a landmark name (e.g., the stopword "and" appears in the restaurant "nice and lovely". We remove these stopwords as we later determine whether a preposition proceeds a landmark, and we consider [preposition] [landmark name] to be equivalent to [preposition] [stopword] [landmark name].

[9] In Nairobi, we found that matatu (minibuses) often were referred to by the location where they traveled to; consequently, we mask phrases such as: "githurai bus", "rongai matatu", machakos minibus", etc. In masking, we replace each word in the phrase with a random sequence of characters. Doing this preserves that a word appears at a location in the tweet, which may affect procedures such as determining the landmark closest to an event word.

[10] This procedure will often capture the same landmarks as captured in the preceding steps; however, it helps to capture other landmarks where the process for augmenting the gazetteers did not generate the landmark name contained in the tweet

(b) Go to the next word in the tweet and further restrict landmarks to those that contain that word. Repeat until doing so would remove all landmarks considered.[11]

(c) Among extracted landmarks, determine which landmark has the smallest number of words and only keep landmarks with that number of words.[12].

---

**C. Extract point locations from roads**

1. For each found, check if the length of the diagonal along the bounding box is less than 500 m; if it is, take the centroid and consider this location to be a landmark[13].
2. If two or more roads are mentioned, find intersections between each road pair. If two roads intersect at multiple locations, only add the intersection if these locations are within 1 km.

---

**D. Restrict landmarks to consider**

1. If the name of a landmark and a road overlap, keep the road and remove the landmark (if a landmark and area overlaps, we keep both).
2. If the name of an exact and fuzzy (misspelled) landmark overlap, keep the exact landmark
3. If a landmark name is contained within another, keep the longer name.

---

**E. Remove landmarks**

1. By roads, areas and tier 1 landmarks

   (a) If a road is mentioned, for each landmark name check if any landmarks with the landmark name are near (within 500 m of) a road. If this is the case, restrict the landmarks in the gazetteer to those that are near the road. If no landmarks are near the road, do not subset and keep the landmark name[14]

   (b) If an area is mentioned (e.g., a neighborhood), for each landmark – follow the same steps as above.

   (c) If a landmark is mentioned after a tier 1 preposition (e.g., "next to", "just after"), for each other landmark – follow the same steps as above, checking the distance between the other landmarks to landmark locations after tier 1 prepositions.[15]

2. Dominant Cluster and "general" landmarks

   (a) For each landmark name, check if the locations form a dominant cluster

[11] For example, in the tweet "accident at garden city toward town", the algorithm searches for landmarks after 'at.' It first finds all landmarks that contain 'garden', then it narrows down these landmarks to those with both 'garden' and 'city'. No landmark contains 'garden', 'city' and 'toward', so the algorithm stops and considers landmarks with 'garden' and 'city'.

[12] For example, if 'garden city', 'garden mall', 'garden city mall' and 'airtel money agent rock city gardens' were extracted, the algorithm keeps 'garden city' and 'garden mall'

[13] These cases are often flyovers and roundabouts

[14] We keep the landmark because during a later step we check for similarly named landmarks near the road, and for the possibility that the extracted road is incorrect, so we still keep the landmark for now).

[15] Helpful in case the landmark near a tier 1 preposition doesn't form a dominant cluster, but a dominant cluster is formed from another landmark mentioned).

i. If they do
   A. keep the landmarks in the cluster and remove the others.
ii. If they don't,
   A. keep landmarks of commonly referenced types (e.g., matatu stages); if a landmark does not contain a common type, don't subset. For this we use the analysis described earlier on determining landmark types more commonly used as crash locations.
   B. Re-check which landmarks don't form a cluster; among these, keep landmarks if the name of the landmark was not derived from an n/skip-gram (ie, matches the original name).[16]

(b) Remove landmark name if it does not form a cluster except if the name follows a tier 1 preposition. (If it follows a tier 1 preposition, it is likely the correct landmark name but just cannot find the exact location; if it does not follow a tier 1 preposition, it is more likely to be a spurious landmark).

[16] For example, if there are 3 landmarks of "garden city", where the original names were: garden city, garden city mall and garden city bank, keep "garden city"; if no name matches the original name, keep all landmarks.

---

**F. Select landmark names or intersections**

1. If there are multiple location names found (eg, multiple landmark names, multiple intersections)
   (a) Loop through preposition tiers. Within each tier, check the following, stopping once a location name has been found.
      i. Check if a landmark name comes after the preposition
      ii. Check if one of the road names used to construct an intersection comes after the preposition
   (b) If no location name has been found, loop through the preposition tiers again and check whether [landmark name] [3 or less words] [preposition name] occurs; if so, keep landmark name(s) with fewest words between name and preposition
   (c) If one intersection found (eg, if 3 or more roads found, and only one pair of roads intersects), use the intersection location.
   (d) Use the landmark closest (least words between) itself and an event word
2. If a landmark name was chosen (ie, not an intersection).
   (a) If multiple landmark names were selected[17]
      i. If a road is mentioned, choose landmarks within 500 m of mentioned road; if none near the road, don't subset

[17] For example, two landmarks in front of different tier 1 prepositions

ii. Choose landmark closest to the event word

(b) If landmark name mapped to multiple locations

i. Select locations within 500 m of mentioned road; if none near road, don't subset

---

**G. [If landmark location is not near any mentioned road] Broaden search to find similarly named landmarks near the road**

1. Start with all landmarks that are near any mentioned road and subset to those that contain the landmark name. Take the next word in the tweet and subset landmarks that contain this word. Repeat process until doing so would cause no landmarks to be found. Among these locations:
   (a) If a dominant cluster exists, use this location.
   (b) If no dominant cluster exists, further subset locations to those where the landmark word in the tweet is at the beginning of the landmarks found. If a dominant cluster is found, use this location.
      i. If no location is found in the previous step, repeat, but check words in the tweet proceeding the landmark name.

---

**H. Snap to Road**

1. If a road is mentioned, snap location to road
2. If no road is mentioned, snap to nearest road if road within 500 m.

---

**I. Final checks to determine whether location should be used**

1. If a road is mentioned and the location chosen is greater than 500 m from any mentioned road, no location is outputted by the algorithm
2. If multiple landmarks are mentioned, the closest landmark to the crashword is used[18] and the landmark is more than two words away from the crash word, no location is outputted by the algorithm
3. If multiple landmarks are mentioned, a tier 5 or 6 phrase precedes the chosen landmark and the landmark is more than two words away from the crash word, no location is outputted by the algorithm

---

[18] This would happen when no tier 1-6 phrase precedes a landmark

## Geoparse Tweets - Full Results

Table S5 shows full results of the geoparsing algorithm. In particular, the table shows the value added of different data sources to build the landmark gazetteer; we run the algorithm using the augmented gazetteer generated from Geonames, Google and OpenStreetMap separately. Results highlight that the algorithm mainly relies on landmarks scraped from Google maps; recall and precision are only slightly worse using Google alone compared to combining all sources. Geonames performs poorly and OpenStreetMap performs better but still worse than Google, achieving about 0.2 and 0.1 worse recall and precision respectively compared to Google when judging whether the algorithm captures the true crash location.

Table S5: Tweet Geoparse Results

| | Any Location Captured by Algorithm Close to True Crash Location | | Crash Location Determined by Algorithm Close to True Crash Location | | Algorithm Cluster Contains True Crash Loction | |
|---|---|---|---|---|---|---|
| | Recall | Precision | Recall | Precision | Recall | Precision |
| **LNEx** | | | | | | |
| LNEx Aug Gaz | 0.674 | 0.686 | 0.129 | 0.132 | 0.175 | 0.125 |
| **Algorithm - by Source** | | | | | | |
| Aug Gaz - Geonames | 0.124 | 0.326 | 0.112 | 0.455 | 0.124 | 0.446 |
| Aug Gaz - Google | 0.79 | 0.853 | 0.645 | 0.811 | 0.653 | 0.777 |
| Aug Gaz - OSM | 0.52 | 0.691 | 0.431 | 0.728 | 0.446 | 0.691 |
| **Algorithm - All Sources** | | | | | | |
| Raw Gaz | 0.695 | 0.757 | 0.579 | 0.756 | 0.591 | 0.72 |
| Aug Gaz | 0.798 | 0.857 | 0.651 | 0.811 | 0.656 | 0.774 |

## Choosing Parameters for Clustering Crash Reports into Unique Crashes

Multiple people often tweet about the same crash. In order to cluster crash reports to unique crashes, we cluster by the kilometer and time distance between reports. To determine optimal kilometer and time parameters, a team manually determined which crash reports refer to the same crash. The dataset was double coded by different team members, resulting in two "truth" datasets. To judge whether crash reports refer to the same crash, the team used the location of the crash, the time of the tweet and looked for details about the crash in the tweet itself (e.g., extent of injuries, types and numbers of vehicles, etc.).

The below table shows summary statistics of the maximum distance and time between any two crash reports in the same clustered or individual crash. Before calculating the statistics, outliers were removed (we define an outlier as a crash cluster where reported crashes occurred over 24 hours or over 5 km from each other). Across both truth datasets, around 52% of tweets were clustered with another tweet, meaning that 48% of tweets are the only tweet reporting one crash.

Table S6: Clustered Tweets Truth Data Summary Statistics

| Variable | Min | Quartile 1 | Median | Mean | Quartile 3 | Max |
|---|---|---|---|---|---|---|
| Truth Dataset 1 | | | | | | |
| Hours Diff | 0 | 0.133 | 0.55 | 1.68 | 1.693 | 23.776 |
| KMs Diff | 0 | 0 | 0.013 | 0.213 | 0.138 | 3.328 |
| N Tweets | 2 | 2 | 2 | 3.324 | 3 | 44 |
| Truth Dataset 2 | | | | | | |
| Hours Diff | 0 | 0.161 | 0.597 | 1.715 | 2.144 | 23.417 |
| KMs Diff | 0 | 0 | 0.024 | 0.276 | 0.199 | 3.13 |
| N Tweets | 2 | 2 | 2 | 3.275 | 3 | 19 |

We examine two common metrics for evaluating clustering performance: the adjusted Rand index and the Jaccard coefficient[9]. When using our algorithm to cluster crash reports, we test all combinations of 0.1, 0.5, 1, 2 and 3 kilometers and 1, 2, 4, 12 and 24 hours. For truth dataset 1, both the Rand index and Jaccard coefficient show that 12 hours and 500 m leads to best results, while truth dataset 2 shows 2 hours and 500 m (see figure ). The difference in results in the truth datasets likely results from the exercise being partially subjective, particularly when limited or no

crash details are provided in the tweet text.

We opt for using thresholds of 500 m and 4 hours. The 4 hour threshold is in between the optimal value from both truth datasets.

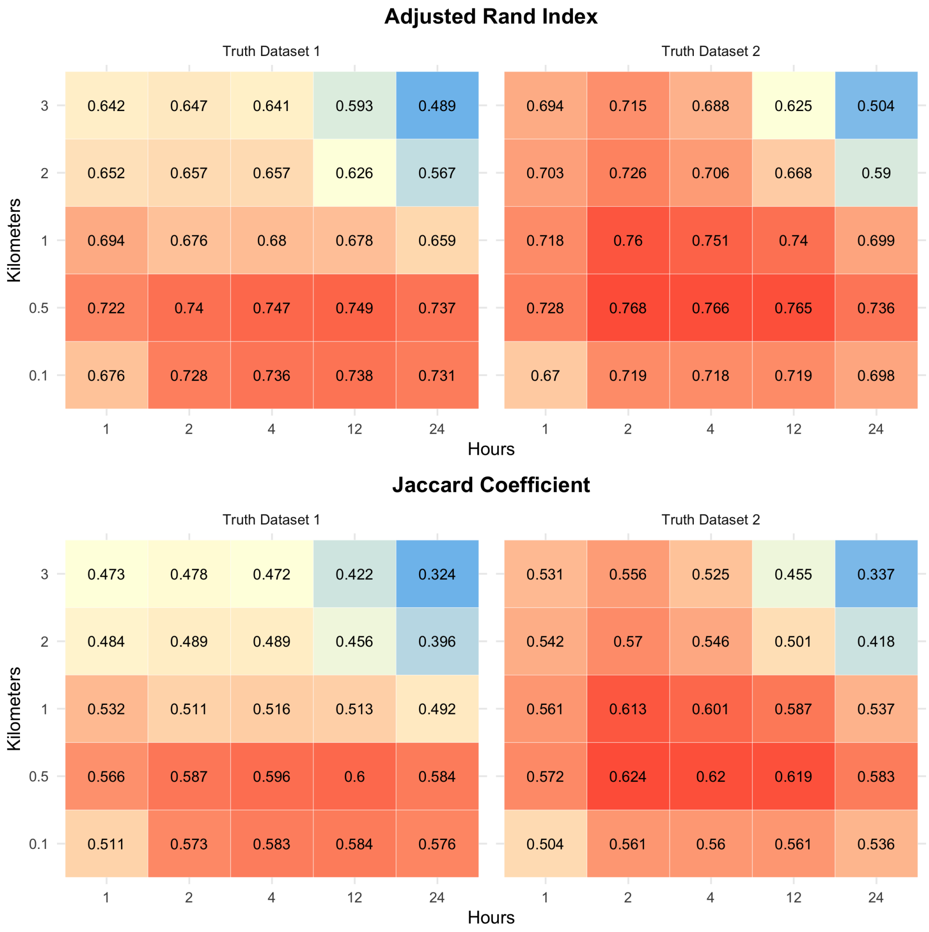


Figure S5: Cluster Evaluation Results

# References

[1] Ritter A, Clark S, Mausam, Etzioni O. Named Entity Recognition in Tweets: An Experimental Study. In: Proceedings of the 2011 Conference on Empirical Methods in Natural Language Processing; 2011.

[2] Gelernter J, Balaji S. An algorithm for local geoparsing of microtext. GeoInformatica. 2013;17(4):635–667. doi:10.1007/s10707-012-0173-8.

[3] Malmasi S, Dras M. Location Mention Detection in Tweets and Microblogs. In: Hasida K, Purwarianti A, editors. Computational Linguistics. Singapore: Springer; 2016. p. 123–134.

[4] Middleton SE, Middleton L, Modafferi S. Real-Time Crisis Mapping of Natural Disasters Using Social Media. IEEE Intelligent Systems. 2014;29(2):9–17. doi:10.1109/MIS.2013.126.

[5] Al-Olimat HS, Thirunarayan K, Shalin V, Sheth A. Location name extraction from targeted text streams using Gazeteer-based statistical language models. Arxiv preprint. 2017;11(17).

[6] Gu Y, Qian ZS, Chen F. From Twitter to detector: Real-time traffic incident detection using social media data. Transportation Research Part C: Emerging Technologies. 2016;67:321 – 342. doi:https://doi.org/10.1016/j.trc.2016.02.011.

[7] Joachims T. Text categorization with Support Vector Machines: Learning with many relevant features. In: Nédellec C, Rouveirol C, editors. Machine Learning: ECML-98. Berlin, Heidelberg: Springer Berlin Heidelberg; 1998. p. 137–142.

[8] Aggarwal CC, Zhai CX. Mining Text Data. Boston, MA: Springer; 2012.

[9] Santos JM, Embrechts M. On the Use of the Adjusted Rand Index as a Metric for Evaluating Supervised Classification. In: Alippi C, Polycarpou M, Panayiotou C, Ellinas G, editors. Artificial Neural Networks – ICANN 2009. Berlin, Heidelberg: Springer Berlin Heidelberg; 2009. p. 175–184.
