# Supplementary material for: Applying machine learning and geolocation techniques to social media data (Twitter) to develop a resource for urban planning
Source: PLoS One. 2021 Feb 3;16(2):e0244317. doi: 10.1371/journal.pone.0244317 (PMC7857609; doi:10.1371/journal.pone.0244317)
Supplement: Attachment — Submitted filename: Response to Reviewers.pdf [file pone.0244317.s002.pdf]

November 12, 2020

PLOS ONE Editorial Board
1160 Battery Street
Koshland Building East, Suite 225
San Francisco, CA 94111

Dear Editorial Board,

Thanks so much for considering our paper for your journal. We are happy to present you with a revision which we believe thoroughly addresses your comments. We also sent the document to an editor to address the clarity of our text; we believe you will find a much improved paper.

It should be noted that this version of the paper we removed a conversation about official data we received from Nairobi's police department. We did this because the police department would not allow us to release the data openly and because it was not essential to our main research question and was only added in order to note that this type of data exists. We did not want to risk the privacy concerns the police flagged as a concern for an element of the paper which was not necessary to illustrate the work we did on our algorithm.

In addition, in figures 2 and 3 we use OpenStreetMap road data. OpenStreetMap is open data and is licensed under the Open Data Commons Open Database License (ODbL) and the CC-BY-SA 2.0 (https://www.openstreetmap.org/copyright). As OpenStreetMaps requires attribution, we have included a sentence in the figure notes that the road data comes from OpenStreetMaps.

In regards to data, we have submitted the Twitter data and the crash verification data to the Microdata catalogue. The datasets are under review and will be released publicly soon. In accordance with Twitter's Terms of Use, we do not include the text of tweets in the Twitter data; however, we include the Tweet IDs, which can be used to extract the text of the tweet with a Twitter API key.

Please see below our response to each of the reviewers comments in turn.

Reviewer #1: This paper applies machine learning and geolocation techniques to social media data (Twitter) to develop a resource for road traffic crashes. The research topic is interesting and worth of investigation. The performance of the proposed methods is demonstrated by a case study conducted in Nairobi, Kenya. The paper is generally well organized and written. **A minor suggestion is that more works on applying machine learning in road traffic crashes are acknowledged in the Introduction section, such as**:
Rule extraction from an optimized neural network for traffic crash frequency modeling. Accident Analysis and Prevention, 2016, 97: 87-95.
Modeling nonlinear relationship between crash frequency by severity and contributing factors by neural networks. Analytic Methods in Accident Research, 2016, 10: 12-25.

Response: Thank you for pointing out this gap in the literature review. We have added both references, which nicely demonstrate the use of machine learning more broadly for road safety analysis, in addition to the following paper:
A comparative study on machine learning based algorithms for prediction of motorcycle crash severity. PLoS One, 2019; 14(4)

Reviewer #2: The topic of this paper is interesting. The methods sound. The results are meaningful and useful. There are several suggestions to improve this paper.

1. "We identify 57,886 crash reports and geolocate 36,428 of them." The percentage of geolocating the crash reports is low. The authors need to clarify this issue.

Response: Thank you for this comment. While 57,886 (now updated in the paper to 52,228) tweets are identified as crash reports, there is a large percentage of them where there is not enough information provided in the tweet to geolocate. In our Truth dataset where tweets were individually coded by a team of local enumerators familiar with the city and landmarks, we found that only 63% of tweets reporting a crash contain enough information to identify an approximate location. If we are to assume the same percentage of tweets contain enough information to be identified in the full dataset of 52,228 crash tweets, then there should be around 32,903 tweets with enough information to be geolocated. This aligns almost perfectly with the number of tweets that the algorithm was able to geolocate (updated in paper to 32,991). We agree that this was not clear in the text and we have added language to provide more clarity.

2. "92% of verified tweets correspond to a crash, demonstrating the validity of crowdsourced crash data." This findings are great. The authors could mention the instructions and future directions for verifying tweets and relating these verified tweets with crash data.

Response: I am glad you liked the method. We have included additional details in the text about the ground truthing process. The details now read:

The Sendy driver was tasked with verifying and reporting whether a crash actually happened in that location. If a driver could not see the crash, they were instructed to ask a bystander whether a crash had occurred but was cleared or whether a crash occurred nearby. Drivers were able to arrive at the crash location quickly; the median time between being alerted of a crash and arriving at the scene was 26 minutes.

Thank you,

Sarah Williams
Associate Professor of Technology and Urban Planning
Director of the Civic Data Design Lab
MIT - Department of Urban Studies and Planning
Tel : 267-253-4124
Email: sew@mit.edu

Contributing Authors
Sveta Milusheva, smilusheva@worldbank.org
Robert Marty, rmarty@worldbank.org
Guadalupe Bedoya, gbedoya@worldbank.org
Arianna Legovini, alegovini@worldbank.org

World Bank
Development Impact Evaluation Department 1818 H Street
Washington, DC 20433

Elizabeth Resor, elresor@ischool.berkeley.edu

University of California Berkley School of Information
102 South Hall # 4600
Berkley, CA 94720
